# Complete Genome Sequence of *Bacillus* sp. Strain RZ2MS9, a Multitrait Plant Growth Promoter

**DOI:** 10.1128/MRA.00623-20

**Published:** 2020-07-30

**Authors:** Maria Leticia Bonatelli, Jie Li, Aline Dumaresq, Maria Carolina Quecine, Matthew L. Settles

**Affiliations:** a Department of Genetics, Luiz de Queiroz College of Agriculture/University of São Paulo, Piracicaba, São Paulo, Brazil; b Bioinformatics Core, University of California, Davis, California, USA; c National Industrial Training Service/Chemical and Textile Industry Technology Center, Rio de Janeiro, Rio de Janeiro, Brazil; University of Arizona

## Abstract

Here, we report the complete genome sequence of *Bacillus* sp. strain RZ2MS9, a plant growth-promoting bacterium isolated from the rhizosphere of guarana, a native crop from Amazonas, Brazil. The assembled genome comprises 5.35 Mbp, no plasmids, and a GC content of 35.22%.

## ANNOUNCEMENT

The use of plant growth-promoting rhizobacteria (PGPR) in agriculture represents a more ecological approach to agricultural practices due to the decreased use of fertilizers and pesticides (1). This is possibly due to a number of mechanisms used by PGPR to promote plant growth, such as solubilization of mineral nutrients (2, 3), production of plant growth regulators (4, 5), acting as biocontrol agents (6), and others. Understanding the mechanisms by which PGPR promote plant growth is important for successful bioinoculant formulation.

*Bacillus* sp. strain RZ2MS9 was isolated from the rhizosphere of guarana, an important Amazon crop (7). This strain showed several *in vitro* plant growth-promoting features, such as nitrogen fixation, phosphate solubilization, and production of plant hormones and siderophores (7). Under greenhouse conditions, *Bacillus* sp. strain RZ2MS9 promoted the growth of maize (Zea mays) and soybean (Glycine max), enhancing shoot dry weights of both crops and root dry weight of maize (7). Due to its importance, a draft genome sequence of this bacterium was previously sequenced (8). However, it is fragmented, consisting of 33 contigs with an *N*_50_ value of 1,097,374 bp. Here, we present the complete continuous genome sequence of *Bacillus* sp. strain RZ2MS9, which can be used to better understand the mechanisms it uses in plant growth promotion.

*Bacillus* sp. strain RZ2MS9 was isolated in 10% tryptone soya agar (TSA) medium (7), and it was grown in Luria-Bertani (LB) broth (10 g · liter^−1^ peptone, 5 g · liter^−1^ yeast extract, 10 g · liter^−1^ NaCl) at 28°C and 150 rpm for DNA extraction. Genomic DNA was extracted with the phenol-chloroform protocol (9). Sequencing was performed with a Pacific Biosciences (PacBio) Sequel sequencer at the National Industrial Training Service/Chemical and Textile Industry Technology Center in Rio de Janeiro, Brazil. The protocol for preparing multiplexed microbial libraries using the SMRTbell express template prep kit version 2.0 (Pacific Biosciences, CA) was followed to prepare the library, and sequencing was carried out in a single-molecule real-time (SMRT) cell (SMRT Cell 1M version 3) tray with a 2-h preextension time and 10 h of movie time.

CCS version 4.0.0 from SMRT Link version 8.0 (Pacific Biosciences) was used to generate highly accurate consensus sequences, and Canu version 1.8 (10) was used to assemble the genome. Arrow version 2.3.3 from the Genomic Consensus package (Pacific Biosciences) and Pilon version 1.23 (11) were used to polish the genome. Pilon used previously sequenced Illumina data (8). Default parameters were used for all software. Two PacBio runs generated a total of 188,742 subreads with an average of 8 subreads per zero-mode waveguide and 246-fold genome coverage.

The final assembled genome consists of 1 chromosome of 5,357,194 bp, no plasmids, and a GC content of 35.22%. The continuity of the *Bacillus* sp. RZ2MS9 genome sequence has been significantly improved over that of the previously published genome sequence (8). Annotation was performed with the NCBI Prokaryotic Genome Annotation Pipeline (PGAP) (12) and revealed 5,468 genes with 5,315 coding sequences and 153 noncoding sequences (42 rRNAs, 106 tRNAs, and 5 noncoding RNAs [ncRNAs]).

### Data availability.

This whole-genome shotgun project has been deposited at GenBank under the accession no. CP049978.1. The version described in this paper is the first version. The raw reads are available under the BioProject accession no. PRJNA343080. The pipeline used in this assembly can be found at GitHub (https://github.com/mlbonatelli/Bacterial-DNA-Assembly).
